# Biological and Environmental Factors Affecting Risk and Resilience among Syrian Refugee Children

**DOI:** 10.20900/jpbs.20210003

**Published:** 2021-02-24

**Authors:** Arash Javanbakht, Anaïs Stenson, Nicole Nugent, Alicia Smith, David Rosenberg, Tanja Jovanovic

**Affiliations:** 1 Department of Psychiatry and Behavioral Neurosciences, Wayne State University School of Medicine, Detroit, MI 48201, USA; 2 Department of Psychiatry, Brown University, Providence, RI 02906, USA; 3 Department of Gynecology and Obstetrics, Emory University School of Medicine, Atlanta, GA 30322, USA

**Keywords:** trauma, development, refugees, immigrants, mental health, family context

## Abstract

More than 21 million people globally are refugees. More than half of these (>10 million) are children, representing a highly vulnerable population. Most children experience high levels of trauma exposure, including war trauma, as well as substantial migration- and resettlement-related stress. These exposures confer risk for mental health problems, including posttraumatic stress disorder (PTSD), but their relative contributions have not been fully explicated. These effects may be modulated by the developmental timing of trauma and stress exposure: childhood trauma and stress are broadly linked to worse health outcomes across the lifespan, but the developmental specificity of these effects remains uncertain. Refugee children typically experience the trauma leading up to displacement (e.g., civil war) which often lasts for decades, and for some, followed by resettlement. Longitudinal studies that follow children through this process can provide unique insight into how these experiences of trauma, displacement, and resettlement during development impact mechanisms of risk and resilience. They can also elucidate how environmental and physiological factors may modulate the effects of trauma and stress. The present study includes two groups of families (parents and their 7- to 17-year-old children): (1) Syrian and Iraqi refugee families who experienced war-zone trauma before resettling in the United States in ~2016, and (2) Arab immigrant families who did not experience war-zone trauma prior to resettlement in the United States in ~2016. We assessed symptoms of anxiety, depression, and PTSD in refugee and immigrant children and parents. Skin conductance responses, a measure of autonomic response, saliva samples for genetic and epigenetic analyses, and information about social and environmental context, including family structure, resources, and neighborhood quality, were also collected. Refugee participants provided data at three time points spanning ~3 years following resettlement in the United States: Wave 1, within 1 month of resettlement, Wave 2, 12–24 months post resettlement, and Wave 3 planned for 24–36 months resettlement. Immigrant participants will provide data once, within 3–5 years after immigration, matching the age of Wave 1. This comparison group enables us to compare mental health and biomarkers between refugees and immigrants. Results of these comparative analyses will provide insight into the impact of war trauma versus other types of trauma and adversity on biomarkers of child mental health outcomes. Results from the longitudinal analyses will address refugee mental health trajectories over time, and, in children, across development. Initial data from Wave 1 showed high levels of anxiety in refugee children, as well as high levels of PTSD symptoms and anxiety in their parents. Together, results from these comparative and longitudinal analyses will provide insight into multiple aspects of trauma and stress exposure in refugees and immigrants, including how the developmental timing of trauma exposure impacts biomarkers and mental health across development. Our assessment of multiple factors affecting childhood mental health following trauma exposure, including familial, neighborhood and social context following resettlement may identify modifiable targets for interventions to support well-being in refugees.

## INTRODUCTION

### Psychopathology in Syrian Refugee Children Exposed to Stress of War and Migration

Syrian and Iraqi refugees are often exposed to war trauma, as well as the stress of migration, life in refugee camps, and resettlement in a new cultural context [1]. Syrian adults living in refugee camps in the Middle East have very high levels of posttraumatic stress disorder (PTSD) (27.2–83.4%) and depression (37.4–43.9%) [2–7]. We have found similarly high rates of possible PTSD (32.2%), anxiety (40.3%), and depression (47.7%) in adult Syrian refugees shortly after resettlement in the United States [8]. Many of these individuals are Shervi, and their children have also experienced substantial trauma and stress [9]. The independent and joint biological effects of exposure to war trauma, migration stress, resettlement, and, for many, parental mental health problems, have not been examined longitudinally in these refugee children.

A rapidly expanding literature has begun to characterize the impact of war trauma in Syrian refugee children. Over 69% of Syrian refugee children living in camps in Lebanon reported exposure to at least one war trauma, and 43.7% experienced four or more. [10]. Rates of PTSD among refugee children range from 10.0–46.8% [11]. Studies from the Middle East and Germany report PTSD rates of 10.5–36.4% and diagnostic levels of anxiety ranging from 7.3–10.5% [12–15]. We have reported high levels of anxiety (53.5%), separation anxiety (76.7%), as well as above-average PTSD rates (5.9% versus 3.9% in a recent epidemiological sample of children in the United States) [16], among 131 Syrian refugee children within the first month of their arrival in the United States [17].

Prior studies indicate that the developmental timing of war trauma exposure impacts mental health outcomes. Older children experience more severe and persistent mental health symptoms following war trauma than younger children [18]. Parental mental health has also been positively associated with mental health symptoms in Syrian refugee children aged 8–18 years, suggesting that family context is an important predictor across development [19]. A study found that parental PTSD symptoms tend to be chronic, and mostly affected by history of trauma exposure and post migration stress. In this work, parental PTSD correlated with children’s emotional problems, and path analysis suggested parental PTSD was associated with harsh parenting leading to conduct, hyperactivity, and emotional problems in children [20]. Children’s mental health may also be modulated by the stress of resettlement in a new cultural and linguistic context [21]. Two studies have linked greater resettlement stress to depression symptoms in children [22,23]. Interestingly, one study found that that this association was specific to immigrant children, and mental health in refugee children with war trauma exposure was less impacted by resettlement stressors. Together, these findings point to a critical role for individual (e.g., developmental status), familial (e.g., parental mental health), and contextual factors in determining risk and resilience in Syrian refugee children after resettlement.

Childhood trauma exposure is, unfortunately, prevalent in all populations, and substantial research is now addressing how this impacts development. In the United States, 20% of children experience [24], and up to 70% have witnessed, serious violence [25]. The negative impacts of childhood trauma are not limited to mental health. Trauma exposure is also associated with poor academic performance [26] and lower verbal IQ [27,28], as well as increased risk for substance use and physical health issues [17,29,30]. These consequences may be long lasting: adults who experienced childhood trauma are more likely to develop PTSD [31,32], anxiety disorders [33], depression [34], and substance use [35,36]. Childhood trauma is also associated with physical health in adulthood, including obesity [37], pain and chronic fatigue [38,39], cardiovascular disease [40], and metabolic syndrome [41]. Most of these studies, however, rely on adults’ retrospective reports of childhood trauma, and few have directly compare the relative impact of specific trauma types (e.g., violence versus natural disasters). This limits our understanding of how the developmental timing and type of trauma exposure impact outcomes across the lifespan.

### Developmental Timing of Trauma Exposure

The prevalence of anxiety disorders increases during late childhood and early adolescence, suggesting that this period may be developmentally critical in identifying individuals at risk for adult psychopathology [42,43]. In a replication of the National Comorbidity Survey, anxiety was 28.8% [44]. Separation anxiety and specific phobias emerge start at an earlier age, and social phobia and generalized anxiety, often emerge in adolescence and adulthood respectively [42,43]. The developmental timing of trauma exposure is important in determination of risk for developing brain and psychopathology [45]. While prospective studies of childhood trauma are limited, a recent study using retrospective recall identified age 10 as the period with highest severity of recalled trauma [46]. Sensitive periods in brain development and physiology also point to the importance of this age as a critical period in which trauma exposure can have long-lasting neurobiological impacts. Studies using structural magnetic resonance imaging (MRI) [47] found that limbic structures such as the amygdala and hippocampus increase between 4 and 18 years of age. Most prior studies of how trauma impacts brain development have examined trauma cross-sectionally between groups of trauma-exposed versus unexposed individuals [48–50], or across individuals with different levels of trauma exposure [51,52]. More longitudinal studies that assess the type and timing of trauma exposures in relation to brain development and mental health are needed.

### Course of Trauma-Related Illness and Changes in Symptom Severity

The effects of trauma, including PTSD, depression, and anxiety symptoms, may fluctuate as a function of environmental stressors and supports, as well as developmental change. Cross-sectional studies cannot capture such dynamic changes in symptoms or biology [53]. For instance, a person who passes the diagnostic threshold for PTSD at one data collection point may not at another point. Similarly, a person who is categorized as “resilient” based on assessment at one time might not at a later time. Adult studies have shown that other trauma sequelae, such as depression and anxiety symptoms [54], and even PTSD subscales [55], also fluctuate over time. These fluctuations have been linked to shifting stress levels [56–59]. This is highly relevant to refugee populations because there are persistent socioeconomic and environmental stressors associated with resettlement in a new country and culture.

There is a growing body of research on mental health trajectories in trauma-exposed children, but interpretation of findings is complicated by heterogeneity in the types, magnitude, and timing of trauma exposure. Many longitudinal studies of post-traumatic symptom trajectories include children exposed to non-violent traumas (e.g., car accidents, injuries, or natural disasters) [60]. A recent review of 27 longitudinal studies of children exposed to trauma (primarily car accidents or accidental injuries), reported that, for most, symptom severity declined over one year [60]. One large study of 548 Chinese 15- to 18-year-old earthquake survivors showed a small decline in PTSD and depression symptoms at between 6 and 18 months after the trauma [61]. It is unclear if these generalize to children exposed to other trauma types, such as war and interpersonal violence, or to children who experience repeated traumas.

We are aware of one longitudinal study of war trauma mental health sequelae in children [18]. This prospective study of war-affected Afghan 11- to 16-year-olds (*N* = 331) identified trajectories of post-traumatic distress symptoms (PTSS) and depression between two time points spanning one year. Results identified four PTSS trajectories—low, declining, rising, and high—and linked these to differences in the type and recency of trauma exposure. An important next step for research with children exposed to war trauma are longer-term longitudinal studies that can assess symptom fluctuations over time in relation to risk and protective factors across multiple developmental periods. In addition, more studies that examine other consequences of trauma, such as anxiety and dysregulated autonomic response, are needed to more fully characterize the impact of childhood trauma [17].

Multiple factors other than trauma may also impact mental health following trauma exposure. One study of Syrian refugee children in Lebanon found that childhood adversities *other than* war trauma exposures were the most robust predictor of PTSD symptoms [10]. Following resettlement, changes in socioeconomic status, family dynamics, cultural context, and other environmental factors necessitate adaptation both by children and parents. For example, our group found that the high separation anxiety (77%) among Syrian refugee children and adolescents [17] was associated with difficulty going to and staying at school. Another study found an association between poverty in Syrian refugee children and working memory deficits [62]. A complexity of refugee mental health is parsing the effects of war trauma from those caused by resettlement stress [1]. One study reported significantly higher anxiety and depression among Arab refugees compared to Arab immigrants resettling in the Southeast Michigan [63]. Additional data on such differences between refugee and immigrant children are needed to begin to understand the relative impacts of war-related trauma and resettlement stress on mental health.

Longitudinal studies that examine multiple aspects of trauma, a wide range of outcomes including biological measures, and environmental factors that may exacerbate or buffer the impact of trauma are critical to advancing developmental science and identifying interventions [64]. This may be particularly important for studies of children exposed to war trauma, as the above longitudinal study reported that children’s recollection of traumatic events declined significantly over a one-year period [18]. In the context of increasing numbers of refugee, displaced persons, and immigrant populations globally, it is critical to understand how children are impacted by war exposure and migration both proximally and over time [65].

### Epigenetics of Trauma Exposure

Recent advances in epigenetics have began to examine the molecular mechanisms of development of psychopathology following trauma, including among refugees [66,67]. Epigenetic modifications lead to changes in gene expression changes in DNA methylation. Typically, increased methylation at gene promoter regions decrease expression of that gene [68]. These changes may be reversible and can be targeted for therapeutic interventions [69].

DNA methylation differences have been associated with psychosocial stress, childhood trauma, PTSD, and war-exposed refugee children [66,67,70–75]. One prior study from our group identified DNA methylation differences associated with direct exposure to violence in children. Higher trauma exposure was associated with accelerated DNA methylation age [76]. This accelerated epigenetic aging is associated with multiple morbidities over the lifecourse [77]. Because changes in DNA methylation accumulate over time, the molecular signature of trauma exposure may be evident substantially before symptoms of a disorder develop. Identification of how the epigenome responds to trauma may allow for early detection and effective intervention.

### Peripheral Autonomic Correlates of Trauma and Stress, and Symptom Change

The PTSD symptoms of hyperarousal and reactivity, including exaggerated startle response, implicate heightened sympathetic nervous system activity in PTSD patients. Measurement of autonomic data such as skin conductance (SC) offers the potential for an objective assessment of some PTSD symptoms. One early approach utilized trauma-related script-driven imagery procedure [78]. The patient is asked to describe their traumatic event which is then transcribed, recorded and played back to the individual during measurement of autonomic activity [79]. A recent meta-analysis [80] of these early studies indicated that SC was the most sensitive measure of hyperarousal and was stable across time [81]. A novel paradigm that builds on script-driven imagery, but is adapted to be used in challenging settings such as home visits, is SC that can be recorded continuously during a standardized trauma interview using mobile devices such as smartphones and tablets connected to electrodes. These new technological advances allow for low-cost and low-burden applications of psychophysiology without specialized training and easy dissemination. We recently used this method to collect SC data in individuals with trauma exposure and found it to be associated with severity of PTSD symptoms [82].

## SPECIFIC AIMS AND HYPOTHESES

In the present study, we will leverage an existing cohort of Syrian refugee children ages 7–17 and their parents to explore longitudinal changes in symptoms of anxiety, depression, and PTSD, along with their epigenetic, autonomic, and environmental correlates. Our previous work established the impact of war trauma exposure and migration stress in this cohort and their parents within one month of their arrival in the United States. Several characteristics of this cohort will enable in-depth analyses that can augment the existing literature. First, the developmental timing of exposure to war trauma and migration/resettlement stress was well-characterized by extensive interviewing of parents and children within one month of resettlement. Second, there is substantial homogeneity in the type of war trauma exposure, and a high degree of trauma exposure. Third, this is a high risk sample: >30% of parents screen positive for PTSD and >50% of children have high anxiety. Fourth, for >90% of children data were parents’ psychopathology data collected from both parents. Fifth, data from siblings is available for most of the sample. We will follow this cohort for two additional years, with annual collection of self-report measures, saliva DNA samples, trauma exposure, and skin conductance response (SCR) as a peripheral marker of sympathetic nervous system arousal. Altogether, we will have 3 years of data available for analysis. In addition, we will conduct a cross-sectional comparison of anxiety and PTSD symptoms, as well as all above-mentioned biomarkers, between Arab refugee and Arab immigrant children in living in southeast Michigan. This will help to determine specific effects of war trauma beyond stress of resettlement. This study will afford valuable insight into how individual, familial, contextual, and developmental factors impact risk and resilience over time in Syrian refugee children resettled in the United States.

## SPECIFIC AIMS OF THE PRESENT STUDY

### Aim 1: Determine the Course of Behavioral and Biological Signatures of Trauma and Stress in at least 100 Syrian Refugee Children over Three Years following Resettlement

Aim 1a: To determine the course of anxiety and PTSD symptoms across years 1 and 2 post-war trauma.

Aim 1b: To determine the SCR correlates of symptom course in children.

Hypothesis 1: Based on our pilot data, we expect to observe that children with higher SCR will show chronically high PTSD symptoms over time.

### Aim 2: Establish the Epigenetic Predictors of Risk and Resilience following Resettlement

Aim 2a: Examine epigenetic age acceleration in refugee children and its association with symptom changes.

Aim 2b (Exploratory): Examine genome-wide DNA methylation changes over the course of three years post migration and their association with symptom course in children.

Hypothesis 2: We expect that accelerated epigenetic aging and epigenetic differences, particularly in glucocorticoid-responsive genes, will be associated with more mental health symptoms over time.

### Aim 3: Determine the Effects of War Trauma Exposure beyond Environmental Stressors of Migration post Resettlement

Aim 3a: Compare severity of anxiety, PTSD, and depression symptoms between 100 refugee children and 100 age matched non-refugee immigrant children, controlling for resettlement stressors.

Aim 3b: Determine the SCR correlates of the differences in symptoms severity between the two groups.

Aim 3c: Determine differences in epigenetic aging between the two groups.

Hypothesis 3: We predict to find higher level of symptoms severity, higher SCR, and accelerated epigenetic aging among refugee children compared to immigrant children.

## METHODS

All procedures were approved by the University Institutional Review Board (1601014566; approval date: 1/18/2020) and align with ethical treatment of human subjects. During the first wave of data collection, families provided consent to be contacted for future research. For children who themselves and their parents agree, provide assent and sign the consent form, children at least 13 years old provided written assent. Participants are informed that at any time they can choose to stop answering the question and participation in research.

### Participants

100 Syrian children ages 7–17 who entered the US as refugees and resettled in Southeast Michigan. We plan to recruit 50 boys and 50 girls selected from the Wave 1 cohort, which included 131 children. We were initially able to collect data on 131 children until the travel ban instituted by the US government stopped our recruitment. Our intention is to have data on 100 children at all time points considering attrition and loss of some participants. We have screened these children, their parents, and siblings within the first month of their arrival in the country and have already presented the data [8,17].

### Inclusion Criteria

Syrian refugee children ages 7–17 on whom we have Wave 1 data, whose parents have agreed to be contacted for future research, and parents and children/adolescents consent to provide data during home or clinic visit.

### Exclusion Criteria

Participants who do not wish to participate or are unable to consent, or their parents do not consent, current active psychosis, or are ward of the court. We will obtain written consent from parents and verbal assent from children. For the control group, we will recruit 100 non-refugee immigrant children from Middle East, ages 7–17 via the same organization that collaborated in the first wave data collection, and local schools with high Middle Eastern children population. Inclusion and exclusion criteria will be the same as refugee children, except immigrant instead of refugee status.

### Home Visit Data Collection

**Figure 1.**
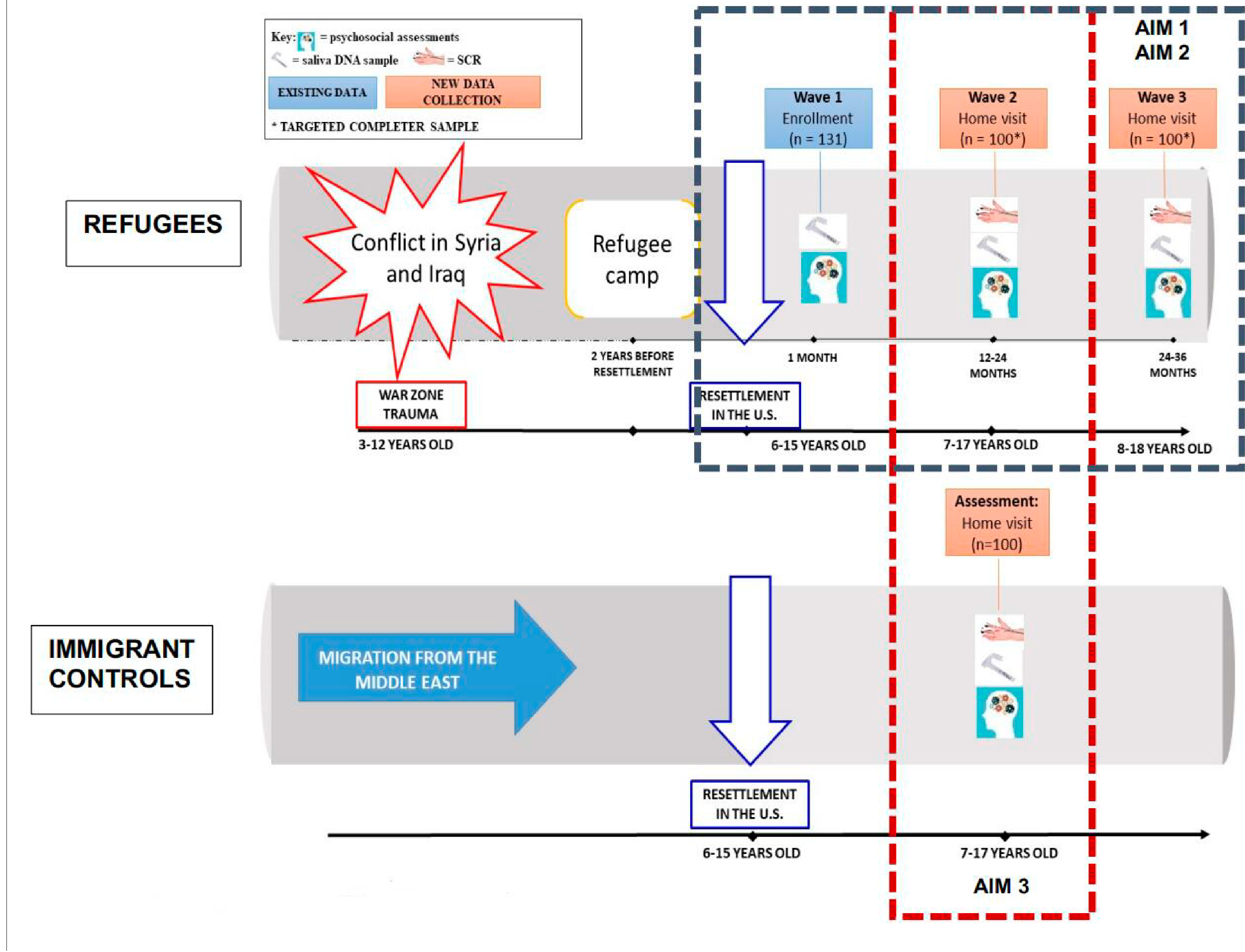
Diagram of Proposed Study Data Collection.

Figure 1 summarizes data collection process. All Wave 2 and Wave 3 data are collected during the home visits, from children and their parents. After consenting, each child participant is interviewed individually by our bilingual research staff, and the questionnaires (available both in Arabic and English) are administered. Adults provide information about environmental stressors related to resettlement. SCR data is collected separately from each child on an iPad in a separated space where possible, and any disturbances in the home will be marked on the data acquisition. Saliva samples for genetics will also be collected using Oragene DNA kits.

### Clinical Assessments

Table 1 summarizes the instruments and the age groups they are used in this study. All of the clinical assessment data are collected at year two and three post migration. Demographic data, screening for PTSD, depression, anxiety, and somatic symptoms in both adults and children are collected by the use of self-reported questionnaires. We also inquire about perceived health and perceived hardship of the whole experience of migration, which during first wave showed a strong correlation with PTSD symptomatology in adults [83]. Perceived social and economic status is measured with MacArthur ladder, and LDQ and PREQIS measures environmental stressors. LDQ includes health services, finances, family, and housing subscales. Our skilled bilingual and bicultural research team members help participants when needed. The data in adults are collected as part of the ongoing refugee studies, but will be available for analyses with the child and adolescent data.

### Epigenetic Data Collection and Analysis

Saliva is collected using the Oragene salivary DNA collection kits (DNA Genotek) as previously reported [84]. DNA is extracted using the automated Beckman Coulter Genomics DNAdvance and Genfind v2 extraction systems, respectively and quantified using PicoGreen (Invitrogen).

The MethylationEPIC BeadChip (Illumina) be used to quantitatively interrogate DNA methylation of >850,000 CpG sites across the genome on 200 samples (i.e., 100 participants × 2 time points, Wave 1 and Wave 2). Data points with detection *p*-values > 0.001 will be considered to be missing data such that CpGs with low confidence scores indicative of measurement error are not included in the dataset. We will exclude any samples with probe detection call rates <95% as well as those with an average intensity value of either <50% of the experiment-wide sample mean or <2000 arbitrary units (AU). We will use hierarchical clustering to identify outliers. We will use “methylumi” to correct for background, assay type, and dye bias. Probes that cross-hybridize between autosomes and sex chromosomes [85] will be removed prior to analysis. For each sample, the proportion of buccal epithelial cells will be estimated, as variation in the relative proportion of these cell types is an important covariate for methylation studies in saliva.

### Skin Conductance Response Measurement

Our preliminary data with PTSD has shown greater SCR to arousing cues [82]. In the current study, we adapted the methodology to measure SCR during the recording of the Infant Crying task described above. The SCR is collected using eSense for iPad (Mindfield Tech, Charleston, USA) using two electrodes connected to the audio input of the iPad. A baseline SC recording of two minutes is collected before the trauma interview. SC data is collected during the HTQ while the child describes the worst trauma experience. As in our studies with adults, a SCR score will be calculated by subtracting the peak SC during the trauma interview from the SC at the end of baseline. Given that this task is low burden and SCR is collected on a mobile device, we are able to administer it during home visits to the refugee families.

### Statistical Analysis Plan

#### Overview and operationalization of variables

The integrated analytic approach adopted here has numerous strengths related to power, reduction of multiple testing, and estimation of *real-world complexity*. Analyses will be conducted using a combination of R [86] and MPlus [87]. Models utilize simultaneous estimation to minimize multiple testing and to match known covariances. Data will be examined for assumptions and maximum likelihood (ML) estimation with robust standard errors (MLR) and related estimation procedures for analysis of non-normal distributions. Consistent with RDoC recommendations for research highlighting “dimensions of observable behavior and neurobiological measures”, analyses will not impose artificial cutoffs but instead examine naturally existing continua. Interpretation will be adjusted for multiple testing to account for false discovery rates [88,89] The use of latent constructs, as compared with data reduction approaches, has the benefit of permitting estimation of unequal weights across indicators, can allow for partial measurement invariance whereby indicators can function differently over time, and growth factor parameters include smaller standard errors and increased power. Risk biomarkers are assessed here as child and adolescent autonomic dysregulation (SCR). Post-trauma symptoms include symptoms of PTSD as well as depression and anxiety. In order to best represent the non-unitary construct of posttraumatic symptomatology, the latent construct of child symptoms will be indicated by symptom severity of common outcomes of trauma (PTSD, depression, anxiety). DNA methylation age acceleration will be calculated using methods described by Horvath [90]. This method accurately estimates age from DNA methylation of 353 CpG sites and was designed to be effective in multiple tissues, including saliva. The CpG sites used to calculate age acceleration are enriched for glucocorticoid response elements and capture stress-related changes in DNA methylation age [91]. The residual between DNA methylation age and chronological age estimates age acceleration, with acceleration observed when participant DNA methylation age is *older* than their chronological age; we will save residuals from a regression of chronological age predicting DNA methylation age, such that over-predicted DNA methylation age residuals reflects DNA methylation age acceleration. As other metrics of epigenetic age are optimized for use in salivary DNA, they will be assessed using the approach described above.

#### Model testing

##### Aim 1. Determine the course of behavioral and biologic signature of trauma and stress in Syrian and Iraqi refugee children.

We expect to observe that anxiety and PTSD symptoms will decrease over time in child and adolescent participants, with symptom levels expected to be coupled to SCR (measured at waves 2 and 3) such that higher levels of PTSD symptoms are associated with higher SCR over time. To test course and coupling of these processes, we capitalize on carefully matched design and theory through latent growth curve modeling (LGM). The intercept will be modeled at baseline (Wave 1) with time parameterized as months post-baseline and with SCR at each wave modeled as a time-varying covariate.

##### Aim 2: Establish the epigenetic predictors of risk and resilience.

For Aim 2a, we expect to see accelerated epigenetic aging associated with greater symptom changes. In order to test the hypothesis that higher child symptoms over time will be associated with DNA methylation age acceleration, we again capitalize on LGM of child symptoms with intercept at baseline and slope parameterized as months post-baseline. We will then examine correspondence of child symptoms and DNA methylation age by regressing DNA methylation age acceleration (the residual of DNA methylation after accounting for chronological age) on the corresponding symptom time point within the growth model and by correlating the two measures of DNA methylation age acceleration; in other words, each DNA methylation age acceleration term will be treated as a time varying covariate, with the hypothesis that we will observe significant associations between symptom severity and DNA methylation age acceleration at these assessments. The benefits of this approach to testing DNA methylation age acceleration (as compared with separately testing DNA methylation age acceleration at each time point in two cross-sectional models) includes improved power afforded by repeated measurement as well as benefits of simultaneous estimation. We also considered modeling the *change* of DNA methylation age acceleration at each wave as related to symptom severity over time. However, by correlating DNA methylation age acceleration measures and modeling symptoms over time in a latent growth framework, the current approach incorporates the autoregressive structure of the data. Next, for our exploratory EWAS (Aim 2b), we expect to see epigenetic changes, particularly in glucocorticoid-responsive genes associated with greater symptom changes. We will characterize child DNA methylation observed in the second two waves of assessment and test for association with posttraumatic symptoms using linear models that test for association of each CpG site and that control for cellular composition, and other relevant covariates such as participant sex. Enrichment of transcription factor binding sites, including glucocorticoid binding, will be examined using oPOSSUM-3, which leverages ChIP-seq data to compute a *Z* score for enrichment of transcription factor binding sites [92]. For all analyses, the false discovery rate will be controlled at 5% to account for multiple testing.

##### Aim 3: Determine the effects of war trauma exposure beyond environmental stressors of migration post resettlement refugee children relative to immigrant children.

In order to test our hypothesis that refugee children will evidence increased severity of anxiety, PTSD, and depression symptoms, even after adjusting for resettlement environmental and parental stressors, we will conduct hierarchical linear regression analyses. Symptom severity outcomes will be modeled in separate analyses, to minimize assumptions about invariance. Demographics will be entered into the first block, followed by resettlement environmental and parental stressors in the second block, and then refugee status into the final block. A similar strategy is used to assess our hypotheses that refugee children, relative to immigrant children, would evidence greater SCR as well as accelerated epigenetic aging (parameterized as residual of DNA methylation after accounting for chronological age). Specifically, we will conduct two hierarchical regressions to model SCR and accelerated DNA methylation aging, again with blocks comprised of (1) demographics, (2) resettlement and parental stressors, and (3) refugee status.

## WAVE 1 FINDINGS

### Wave 1 Symptom Severity Data among Children

Wave 1 data collection took place at Arab American Chaldean Council (a local non-profit) primary care clinics in Detroit Metro Area within the first month of Syrian refugees’ arrival in the US, and during a mandatory health screening visit. 87.7% of the Syrian refugees at the clinics participated. In children, the Screen for Child Anxiety Related Disorders (SCARED) was used to measure anxiety, and UCLA Posttraumatic Stress Reaction Index was used for PTSD symptoms. Mood and Feelings Questionnaire (MFQ) was used to measure depression. Descriptive data related to this cohort of children has been published [17]. 131 Syrian children ages 6–17 (40.5% girls; mean age 11.02 (3.32), 93% accompanied by 2 parents, 85% have at least one sibling available) were recruited. Few had existing medical condition (8.3%) and fewer any existing psychiatric condition (6.5%: 2.8% reported anxiety, 1.9% reported panic attacks, and 1.9% reported depression). Likewise, self-reported lifetime use of tobacco or illegal drugs was rare (1 child). Overall, 53.5% reported their health status as excellent and no one reported that it was fair or poor. English proficiency was limited: 42.6% said they could not speak English at all and 49.5% said they could speak it but not well. Gender differences, apart medical condition (Fisher’s Exact Test, *p* = 0.048), were minimal and not significant. Prevalence of possible anxiety disorder (53.5%, 95% CI 42.7–64.2), separation anxiety (76.7%, 95% CI 67.5–85.6), and PTSD (5.9%, 95% CI 0.2–12.3) was high. At this initial visit, none of the girls but 9.1% of the boys met the criteria for PTSD however, both sexes reported symptoms (mean girls’ UCLA PTSD score = 14.01, boys’ UCLA PTSD score = 18.03) which increased at future visits. The correlation was high between PTSD symptoms and anxiety symptom severity (*r* = 0.65, *p* < 0.001). Symptom severity of separation anxiety was negatively correlated with age (*r* = −0.35, *p* = 0.001).

### Wave 1 Correlations between Parents' and Children's Symptoms

We examined correlations between symptom severity in children, and those of each of their parents in Wave 1. Possible diagnoses of anxiety, depression, and PTSD were increased among mothers (63.6%, 95% CI 46.3–81.0%; 63.3%, 95 CI 45.0–81.6; 41.5%, 95% CI 25.7–57.2, respectively) and fathers (34.4%, 95% CI 17.0–51.8; 40.0%, 95% CI 21.4–58.6; 35.9%, 95% CI 20.1–51.7, respectively). Maternal, but not paternal, PTSD (β = 0.33, *p* < 0.001), anxiety (β = 4.66, *p* = 0.041), and depression (β = 7.37, *p* = 0.003) symptom severity were associated with anxiety symptom severity in the child [17].

### Skin Conductance Data

**Figure 2.**
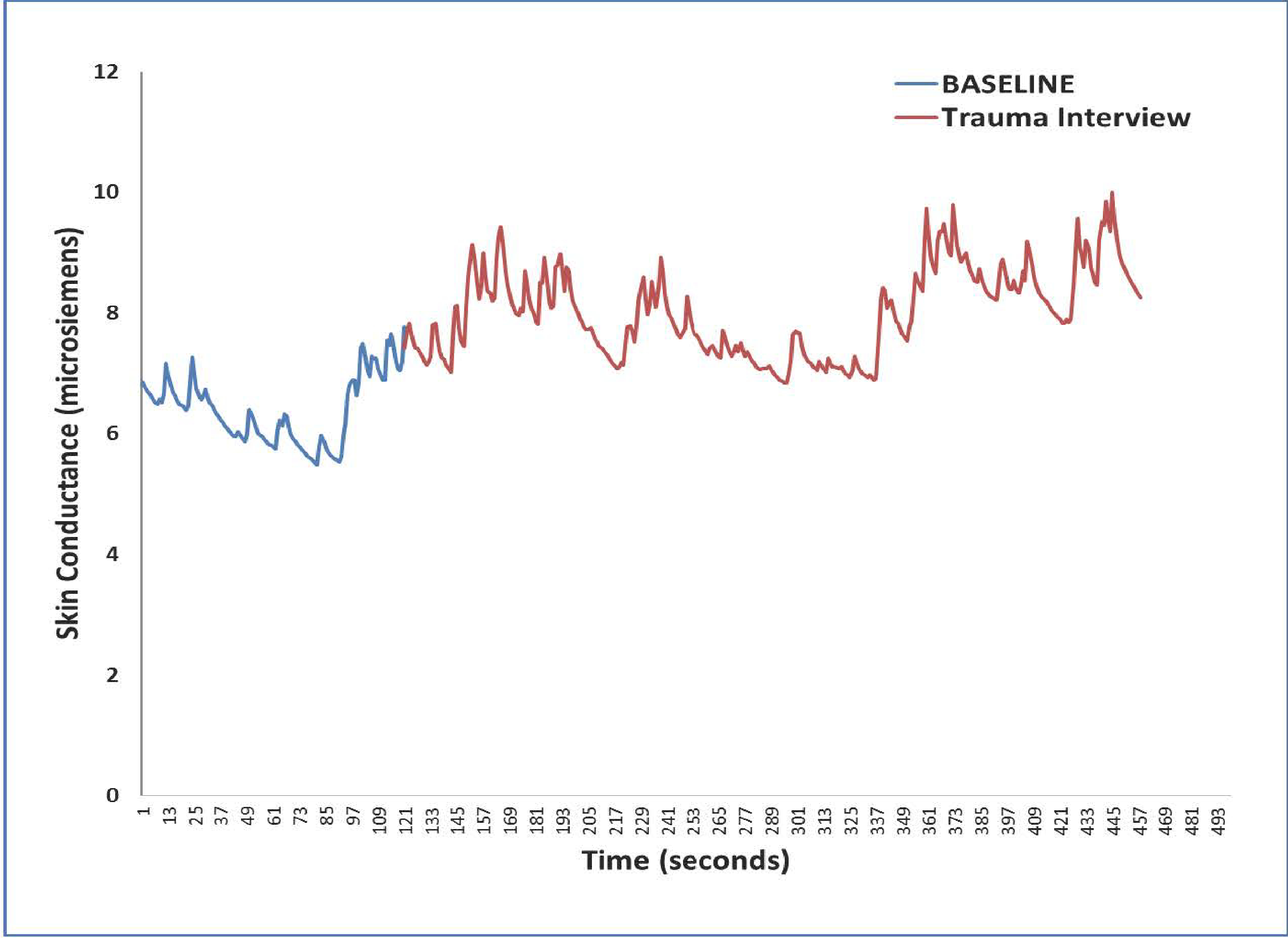
SCR using eSense with a Syrian refugee during a trauma interview.

Our recent prospective PTSD studies of adults recruited from the Emergency Department collected skin conductance response using the same mobile technology proposed in the current application. In a sample of 75 adults from the Grady Trauma Project in Atlanta, we found that SCR strongly predicted PTSD symptoms 6 months after trauma exposure. In order to determine feasibility of using this method with refugee children, we have collected SCR with the mobile app while administering the Harvard Trauma Questionnaire to children. These preliminary data showed: (1) that SC was highly responsive to trauma narrative (Figure 2), and (2) that the children found the questioning with eSense acceptable.

## PRELIMINARY WAVE 2 FINDINGS

### Wave 2 Changes in Symptoms Severity after One Year

Our bilingual/bicultural team has collected pilot follow up data assessing refugee families during home visits one year after arriving in the US. Families in general have been welcoming and data collection has been more successful than the first wave with less missing data. That is because we do not have the time constraints of the first wave. We collected data on parents for 92.6% and sibling data for 96.3% of the pilot sample of children.

**Figure 3.**
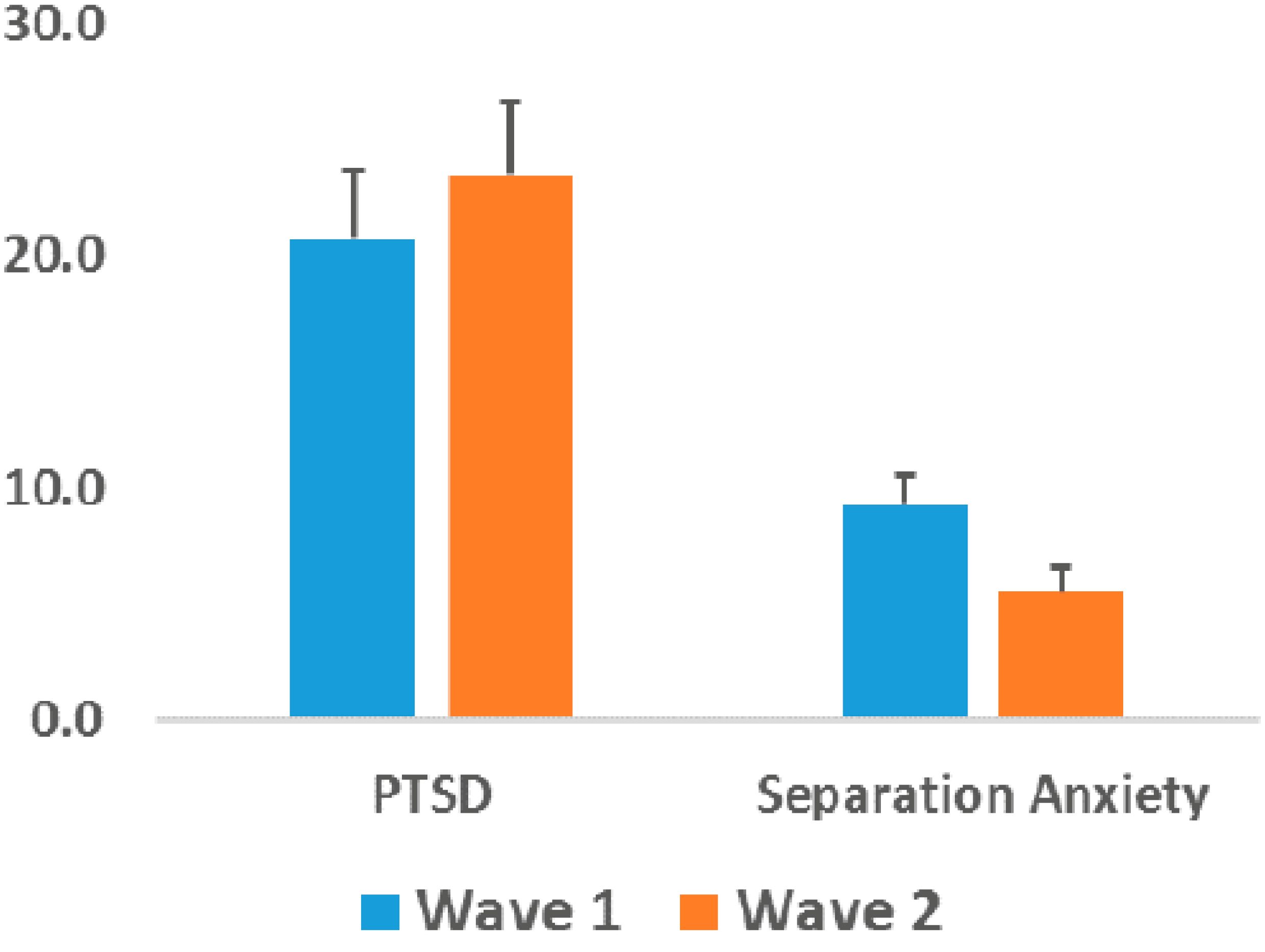
Symptom severity changes from Wave 1 to Wave 2.

Our pilot follow-up data on 39 children suggest a decline in anxiety symptoms, but not PTSD symptoms (Figure 3). For 76.2% of children anxiety symptoms decreased. There were significant declines in the generalized (*p* = 0.019), separation (*p* < 0.001), and social (*p* < 0.001) anxiety subscales. While total anxiety score improved for 76.2% of the children, 19% still screened positive for anxiety at Wave 2. In contrast to anxiety, PTSD symptoms severity increased for 62.5% of children. PTSD symptoms increased from Wave 1 to Wave 2 when controlling for changes in anxiety (*p* = 0.01).

### Effects of Trauma on Epigenetics

**Figure 4.**
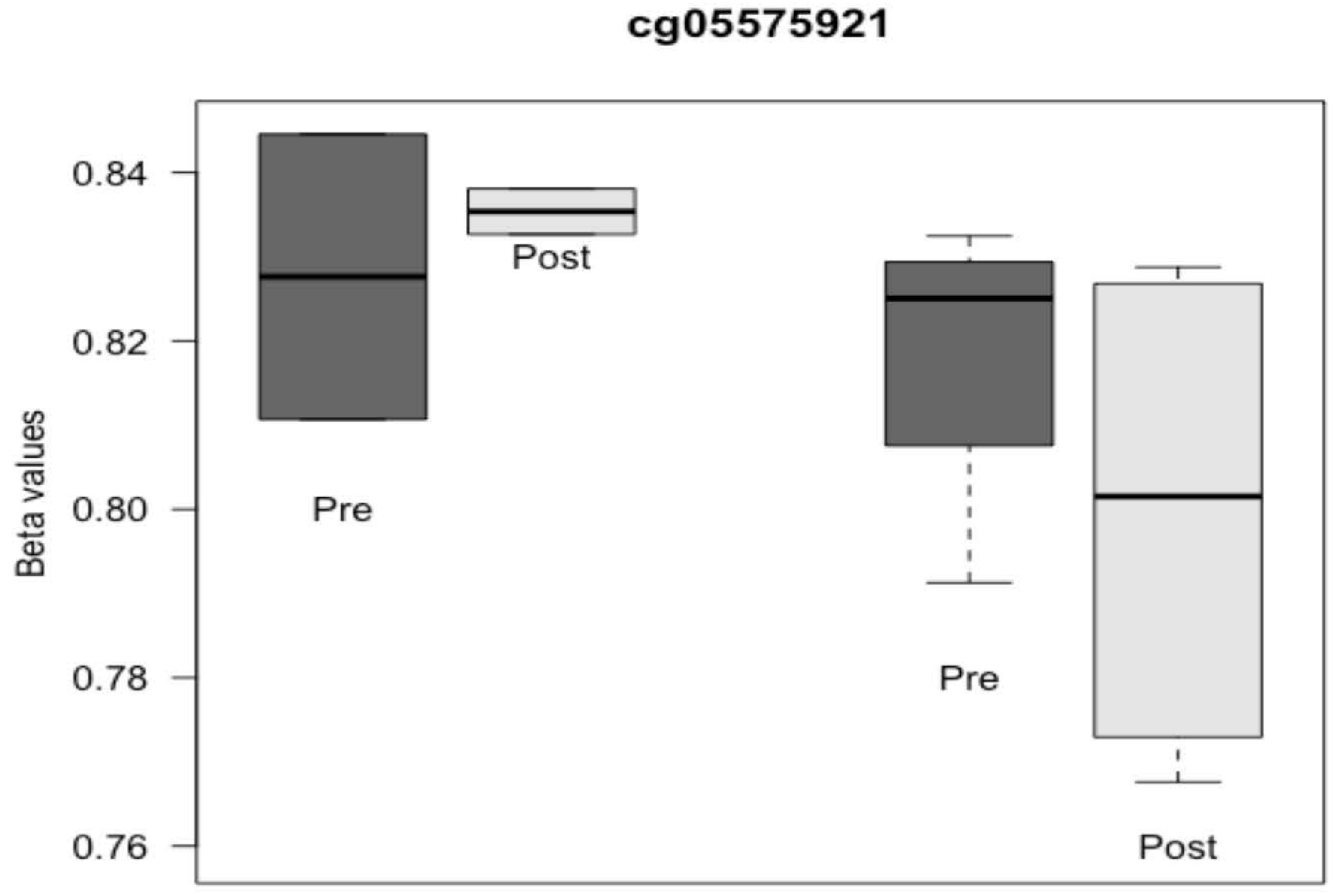
DNA methylation varies over time and associates with post-migration anxiety.

For preliminary analyses, saliva DNA was extracted from 22 refugee cohort subjects, and methylation was interrogated using the MethylationEPIC BeadChip. DNAm age was highly correlated with chronological age (*r* = 0.74; *p* < 0.001), using Horvath’s tissue-independent method. Age acceleration had excellent variability and ranged from −5.07 to 5.24 years. To assess variation in post-traumatic stress symptoms related to individual CpGs, we compared those with and without high anxiety symptoms, controlling for age, gender, and proportion of buccal epithelial cells. Despite the modest sample size, we found multiple CpGs associated after FDR correction at 5%. We also evaluated consistency with our recent meta-analysis of DNAm in the PGC PTSD workgroup [93]. Of the top 10 CpG sites identified (FDR < 0.05), 5 (50%) associated with anxiety in these refugee children (*p* < 0.05) in the same direction as adults from the meta-analysis. For example, methylation of cg05575921, the CpG most associated with PTSD in the PGC meta-analysis (*z* = 6.58; *p* = 4.72 × 10^−11^), is higher in those that report more post-migration anxiety symptoms but substantially lower in those without post-migration anxiety (*t* = 3.44; *p* = 0.0063; Figure 4).

## DISCUSSION

This is the first longitudinal prospective study of trauma exposure, development, and biomarkers in Syrian refugee children over 3 years following resettlement in the United States. Our pilot findings suggest that while the anxiety symptoms may show some decline one year post settlement, trauma symptoms do not seem to decline as an effect of time. We were also able to detect varying methylation between two data collection among the pilot sample. Our approach integrates epigenetic, psychophysiological, environmental, and self-report clinical data to further characterize the etiology of anxiety disorders following trauma exposure. Further, we will specifically examine the differential effects on biology and behavior in relation to developmental processes, family context, resettlement stress, and, potentially, post-resettlement trauma exposure. Our comparison of refugees with comparable immigrant population will facilitate identification of the effects of war trauma versus the stress of migration and resettlement. Instead of treating trauma and stress-related symptoms as static conditions, we will explore biological and environmental factors affecting changes in symptoms severity. This research will significantly contribute to the development of empirically grounded early intervention strategies that may ultimately increase resilience in this population.

## Figures and Tables

**Table 1.** Instruments and the age groups used in this study.

Instrument	Measurement	Adults (18 years old or older)	Refugee Children	Immigrant Children
Demographic Questionnaire	Self-report of sociodemographic information, including age, marital status, religion, country and city of origin, occupation, education status, perception of current health and perceived hardship experienced, medication use, current medical and psychiatric diagnoses, and substance use	×	×	×
Hopkins Symptom Checklist (HSCL-25)	25-item validated measure of anxiety and depressive symptoms. The total score has been shown to be highly correlated with severe emotional distress within refugee populations [78].	×		
Life Events Checklist (LEC)	Self-report measure in which participants indicate traumatic events they have experienced, witnessed, encountered due to work, or learned about; consists of 16 events commonly known to result in the presentation of PTSD symptomatology [79].	×	×	×
McArthur Ladder	Pictorial representation of a ladder upon which participant designates perceived standing economically and socially within his/her community; shown to indicate social and socioeconomic status [80].	×		
Modified Harvard Trauma Questionnaire (HTQ)	Measure specifically tailored toward refugee experiences in which participants indicate verbally whether they have experienced certain traumatic events and expand upon the worst events that have happened to them (Part I and II) [81]; modified for our population.		×	×
Modified Living Difficulties Questionnaire (LDQ)	Self-report measure in which participants indicate how much of a problem they perceive certain post-migration living difficulties to be in their lives [82]; modified to be more appropriate for our population. Subscales include health services, finances, family, and housing.		×	×
Mood and Feelings Questionnaire (MFQ)	Series of phrases describing how frequently the participant may have felt or acted in a certain way within the last two weeks as a measure of depressive symptoms [83].		×	×
PTSD Checklist Civilian (PCL-C; DSM V)	Self-report, 20-item questionnaire assessing PTSD based on DSM criteria; civilian version uses wording and phrasing more appropriate for a civilian audience to screen for symptoms in a non-military population [84].	×		
Perceived Residential Environment Quality Indicators and Neighborhood Attachment	Self-report measure of 11 scales measuring perceived quality of the participant’s living environment and one scale measuring participant’s attachment to his/her neighborhood [85].	×		
Pubertal Development Scale	10 item self-reported questionnaire is a continuous measure of pubertal status [86].		×	×
Screen for Child and Anxiety Related Disorders (SCARED)	41-item self-report inventory used to screen for signs of anxiety disorders in children; measures five domains: panic/somatic, separation anxiety, generalized anxiety, social phobia, and school avoidance [87].		×	×
Somatic Symptoms Scale (SSS-8)	Brief, 8-item scale assessing the somatic burden of common physical pains in the participant’s daily life, common in psychiatric illness [88].	×	×	×
UCLA Child/Adolescent PTSD Reaction Index	Self-report measure that assesses the child or adolescent’s trauma history as wed as potential diagnosis for PTSD based on DSM criteria [89].		×	×
US Household Food Security Survey Module	Self-report six item measure that assesses household food access and security [90].		×	×
